# IL-33 as a Marker of Poor Early Response in Neuroendocrine Tumor Patients Undergoing Peptide Receptor Radionuclide Therapy

**DOI:** 10.3390/ijms26178526

**Published:** 2025-09-02

**Authors:** Katarina Vuleta Nedic, Nevena Gajovic, Ivan Jovanovic, Milena Jurisevic, Marina Jovanovic, Slobodan Jakovljević, Bojana Popovic, Jelena Djordjevic, Vesna Ignjatovic, Vladimir Vukomanovic

**Affiliations:** 1 Department of Nuclear Medicine and Clinical Oncology, Faculty of Medical Sciences, University of Kragujevac, 34000 Kragujevac, Serbia; kvuleta87@gmail.com (K.V.N.); jeladj997@gmail.com (J.D.); vukomanovic@gmail.com (V.V.); 2 Department for Nuclear Medicine, University Clinical Center Kragujevac, 34000 Kragujevac, Serbia; 3 Center for Molecular Medicine and Stem Cell Research, Faculty of Medical Sciences, University of Kragujevac, 34000 Kragujevac, Serbia; ivanjovanovic77@gmail.com (I.J.); milena.jurisevic13@gmail.com (M.J.); marina_jovanovic@rocketmail.com (M.J.); jakovljevicslobodan@gmail.com (S.J.); 4 Faculty of Medicine, University of East Sarajevo, 73300 Foca, Bosnia and Herzegovina; 5 Department of Clinical Pharmacy, Faculty of Medical Sciences, University of Kragujevac, 34000 Kragujevac, Serbia; 6 Department of Otorhinolaryngology, Faculty of Medical Sciences, University of Kragujevac, 34000 Kragujevac, Serbia; 7 Department of Surgery, University Clinical Center Kragujevac, 34000 Kragujevac, Serbia; 8 Clinic of Endocrinology, Diabetes and Metabolic Diseases, University Clinical Centre of Serbia, 11000 Beograd, Serbia; popbojana@gmail.com

**Keywords:** neuroendocrine tumors, peptide receptor radionuclide therapy, IL-33, sST2

## Abstract

Despite a high disease control rate in the treatment of unresectable or metastatic well-differentiated, somatostatin receptor-positive neuroendocrine tumors (NETs) with peptide receptor radionuclide therapy (PRRT), a certain percentage of patients will experience an unfavorable outcome. Besides clinical, hematological, and biochemical parameters, including widely used inflammatory markers, as well as literature-recognized inflammatory indices, there is a growing need for the identification of novel biomarkers as prognostic factors of therapeutic response. In this prospective single-center study, 51 NET patients treated with PRRT were included and divided into two groups: responders and non-responders in accordance with therapeutic outcome. Cytokine, clinical, and biochemical data were analyzed. Non-responders had significantly higher serum concentrations of IL-33 and IL-4 in comparison to responders, while sST2 was increased in responders. A positive correlation was measured between IL-33 and IL-4, as well as between IL-33 and disease progression. A negative correlation was noted between IL-33 and the neutrophil count %. ROC curve analysis identified values of IL-33 >146.5 pg/mL as a predictor of poor early therapeutic response, and logistic regression confirmed its independent prognostic value. Elevated IL-33 and IL-4 favor the development of a type 2 immune response associated with unfavored therapeutic outcome, while increased sST2 mitigates the IL-33’s effect in responders, contributing to a more favorable response. These findings emphasize IL-33 as an important biomarker of early response in NET patients undergoing PRRT.

## 1. Introduction

Neuroendocrine tumors (NETs) are a rare, biologically and clinically heterogeneous group of neoplasms characterized by their capacity for the synthesis, storage, and secretion of various neuroamines and peptides, occasionally resulting in secretory syndromes. However, the majority of neuroendocrine neoplasms are nonfunctional and can remain clinically silent for extensive periods, delaying the diagnosis. Predominantly arising from the gastrointestinal and bronchopulmonary systems, these tumors have shown an increase in incidence over the past three decades [1,2]. Peptide receptor radionuclide therapy (PRRT) has been established as an effective treatment option for unresectable or metastatic neuroendocrine tumors (NETs) in the last twenty years. By targeting tumor cells through binding to widely expressed somatostatin receptors (SSTRs), PRRT enables selective radiopharmaceutical delivery and radiation-induced cell death. Reliable randomized clinical trials demonstrated its favorable safety profile while achieving disease control in most cases [3]. Nevertheless, a considerable number of NET patients undergoing PRRT will experience disease progression [4].

Numerous studies have focused on identifying factors predicting disease progression and treatment outcomes during PRRT. These factors primarily include specific clinical parameters, blood-based biomarkers, and morphological or functional imaging [5].

Since immune mediators affect not only tumor cell proliferation, survival, angiogenesis, and metastasis, but also the response to the various therapeutic modalities, several studies have investigated the role of inflammatory markers as predictive factors in patients with NETs treated with PRRT [6,7,8]. While limited studies have pointed out the predictive impact of a small subset of inflammatory markers, such as C-reactive protein (CRP), composite index as Platelet × CRP multiplier (PCM), CPR/albumin ratio, absolute neutrophil count, and platelet-to-lymphocyte ratio (PLR), further research is required to improve and identify novel inflammatory biomarkers, including cytokines [7,9]. Cytokine profiling may offer valuable insights into the biological response during PRRT and help identify specific cytokines as potential biomarkers of PRRT efficacy.

IL-33 is a cytokine that may play a dual role as a pro- or anti-tumorigenic mediator. Increased IL-33 in serum or tumor tissue may correlate with poor prognosis in several types of cancers, while, on the contrary, IL-33 can also stimulate anti-tumor immune response and thus contributes to tumor cell elimination [10]. In contrast, its soluble receptor sST2 acts like a decoy receptor, with an attenuating effect on IL-33 signaling [11]. Recent studies point to the role of the IL-33/sST2 axis as a valuable immunoinflammatory biomarker in cancer [12]. Alongside this axis, some studies suggest the complex interaction between IL-33 and IL-4 that has a well-established regulatory role in the cancer microenvironment associated with cell survival, proliferation, and metastasis [13,14].

The main aim of this study was to analyze the relationship between clinical, hematological, and standard biochemical parameters, as well as cytokine profile and PRRT outcome.

## 2. Results

### 2.1. Demographic and Clinical Features of NET Patients

A total of 51 patients were enrolled in this study, with a mean age at the time of treatment of 58 ± 1.7 years (range: 30–83 years). Of these, 22 patients (43.1%) were male and 29 patients (56.9%) were female. All patients had a good performance status—ECOG 0–1. The most common primary sites of NETs were Gastroenteropancreatic (GEP-NETs), present in 37 patients (72.5%), followed by unknown primary NETs in 7 patients (13.7%), lung-NETs in 4 patients (7.9%), and others (5.9%). According to interim multidetector computed tomography (*MDCT*) or magnetic resonance imaging (*MRI*), disease control was achieved in 8 patients (15.7%) with partial response (PR) and 29 patients (56.9%) with stable disease (SD). Disease progression was observed (PD) in 14 patients (27.4%). Patients with PR and SD are classified as responders (72.6%), whereas those with PD were considered non-responders (27.4%). PRRT was administered in combination with long-acting somatostatin analogs (SSAs) in all patients except one. Other patient characteristics are detailed in Table 1.

### 2.2. Laboratory Analyses of NET Patients

Significant differences in laboratory parameters were found between the group of responders and non-responders. In the non-responder group, the decrease in white blood cell count (*p* = 0.013, *p* = 0.04), neutrophil count (*p* = 0.037, *p* = 0.039), lymphocyte count (*p* = 0.03, *p* = 0.03), monocyte count (*p* = 0.002, *p* = 0.029), platelets (*p* = 0.021, *p* = 0.014), SIRI (*p* = 0.021, *p* = 0.049), SII (*p* = 0.026, *p* = 0.049) and vitamin D (*p* = 0.037, *p* = 0.029) were noted both before initiation of PRRT and prior to the second cycle, compared to the responder group. Additionally, significantly higher values of AST (*p* = 0.039), ALT (*p* = 0.029), CgA (*p* = 0.036), and 5-HIAA (*p* = 0.03) were measured in non-responders than in responders at the second time point. There were no statistically significant differences in other laboratory parameters between the two groups (Table 2).

### 2.3. NET Patients with Disease Progression Had Elevated Systemic Level of IL-33

**Figure 1:**
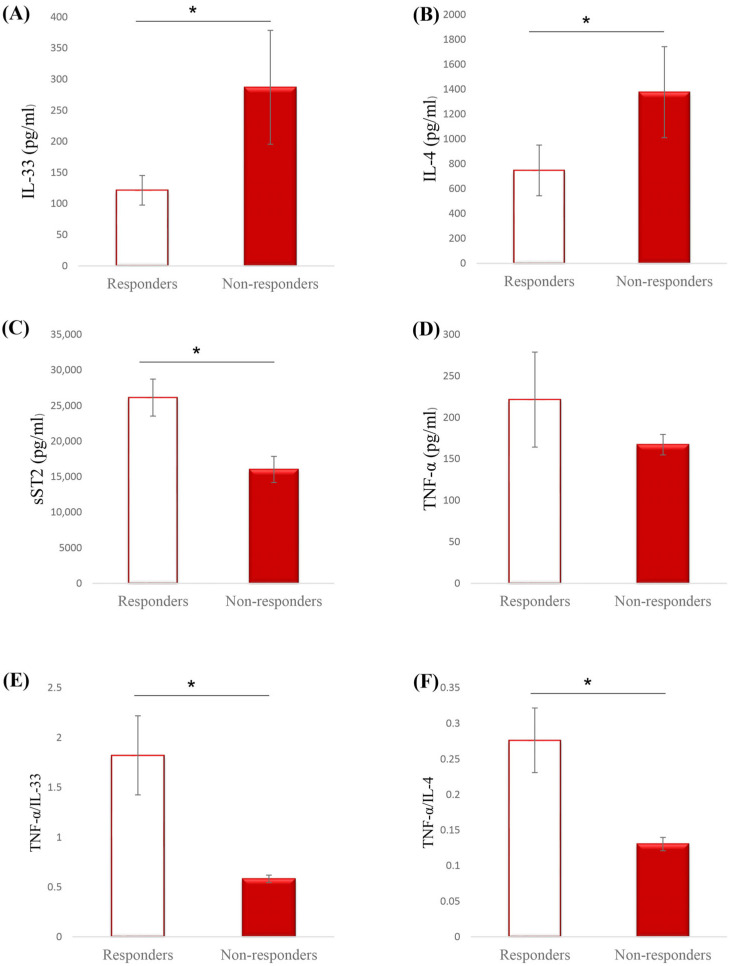
Systemic values of cytokines and ratios of interest. Based on the therapeutic response, all patients were divided into two groups: responders and non-responders. (**A**) Serum IL-33 concentrations (pg/mL), (**B**) Serum IL-4 concentrations (pg/mL), (**C**) Serum sST2 concentrations (pg/mL), (**D**) Serum TNF-α concentrations (pg/mL), (**E**) Ratio of TNF-α to IL-33, (**F**) Ratio of TNF-α to IL-4. Systemic levels of IL-33, IL-4, sST2, and TNF-α were measured by ELISA. Statistical significance was tested by Mann–Whitney U test; * *p* < 0.05 was considered statistically significant.

Serum levels of pro- and anti-inflammatory cytokines were measured in patients with stable and progressive disease. Systemic values of IL-33 (*p* = 0.001) and IL-4 (*p* = 0.001) were significantly lower in responders in comparison to patients with a more severe form of disease, while soluble ST2 (*p* = 0.001) was significantly higher in responders (Figure 1A–C). Although serum level of TNF-α was higher in responders, this alteration did not reach statistical significance (Figure 1D). However, for the purpose of insight into the relationship between counterregulatory cytokines, analyses of ratios of TNF-α with IL-33 and IL-4 were performed. Results revealed that TNF-α/IL-33 (*p* = 0.001) as well as TNF-α/IL-4 (*p* = 0.001) ratios were significantly increased in responders in comparison to non-responders (Figure 1E,F).

### 2.4. IL-33 Correlates with the Disease Progression

As IL-33, together with sST2, were the most prominent among other measured cytokines, we further correlated these two mediators with other cytokines and clinical and laboratory parameters. Strong positive correlation was detected between IL-33 and IL-4 (*p* = 0.001). Moreover, IL-33 positively correlated with disease progression (*p* = 0.037), while a negative correlation was measured with neutrophil count % (*p* = 0.041) (Table 3). sSt2 showed positive correlation with absolute neutrophil count (*p* = 0.022) and gamma glutamyl transferase (*p* = 0.033), while negative correlation was noted between sST2 and triglyceride (*p* = 0.049) and lipase (*p* = 0.001), respectively (Table 3). The ROC curve analysis of IL-33 to differentiate better responders and worse responders to therapy detected that the optimum cut-off point for IL-33 was 146.5 pg/mL (AUC 0.689; 95%CI 0.525–0.853; Sensitivity 64.3%; Specificity 70.3%; *p* = 0.039).

The univariate logistic regression analysis showed that Ki67(>10%) (OR 4.255, 95% CI 1.159–15.625; *p* = 0.029) and IL-33 (>147 pg/mL) (OR 4.552, 95% CI 11.159–15.628; *p* = 0.029) were significant risk factors for disease progression (Table 4).

## 3. Discussion

The aim of this study was to assess the association between clinical characteristics, laboratory parameters, and immune cytokine profile with PRRT outcome. NET patients were divided into two groups (responders and non-responders) according to the criteria described in the Materials and Methods section.

Concerning clinical parameters, no significant differences were observed between responders and non-responders (Table 1). While some reports suggest that younger patients have a better response to PRRT [15], others did not reveal significant differences in outcome regarding age and gender, supporting our findings [16,17]. The prognostic significance of primary tumor location in neuroendocrine neoplasms has been well established in epidemiological research [1]. Consistent with the previous study [18], our analysis also demonstrated that GEP-NETs were predominantly represented among both responders and non-responders compared to lung-NETs, unknown primary NETs, and other primary sites, although this did not reach statistical significance. Nevertheless, further studies with larger cohorts are needed to fully assess the efficacy of PRRT in non-GEP-NETs in comparison to GEP-NETs.

In addition, our study revealed significantly lower baseline and intratherapeutic values of white blood cells, neutrophils, lymphocytes, monocytes, platelets, SIRI, SII, and vitamin D in the non-responder group.

Some previous studies have shown that tumor progression is promoted by chronic inflammation, resulting in poorer PRRT outcomes. Increased levels of inflammatory markers such as neutrophils, CRP, CRP/Alb, and composite indices like NLR, PLR, SIRI, and SII have been associated with more aggressive tumor behavior, immunosuppressive tumor microenvironment, and limited therapeutic efficacy [7,8,9,10,19]. Contrarily, this study found that a decreased number of neutrophils, lymphocytes, monocytes, and platelet counts may imply that the immune system is potentially compromised or myelosuppressed, leading to ineffective anti-tumor responses and PRRT’s reduced efficacy. Vitamin D levels were also significantly reduced in non-responders before the start of PRRT and before the second cycle. Interestingly, recent work of Modica et al. demonstrated that patients with lung NETs and bone metastases had vitamin D insufficiency, with significantly lower vitamin D levels compared to patients with lung NETs without bone metastases, suggesting a possible role of hypovitaminosis D in bone metastasis development and, thereby, disease progression [20]. These findings support previously published data about the role of vitamin D as an immunomodulator in the context of tumor immunity [21].

The enzymes AST and ALT are standard parameters in the assessment of liver function. While ALT is specific for hepatocytes, AST is expressed in other tissues, such as skeletal muscle, cardiac muscle, and kidney [22]. Therefore, elevated AST values without a concomitant increase in ALT may imply extrahepatic pathology rather than primary liver damage [23]. However, increased AST levels in non-responders undergoing PRRT, as demonstrated in our study, may indicate an altered tumor cell metabolism, specifically, anaerobic glycolysis, known in the literature as the “Warburg effect” [24]. This metabolic adaptation modulates the activity of AST, since AST is needed for the maintenance of high proliferation rates of cancer cells [25].

Higher ALP levels are associated with worse outcomes in several cancers and may act as a prognostic marker, particularly in patients with liver or bone metastatic disease [26,27]. Moreover, a recent prospective study indicates that ALP may serve as a strong prognostic marker in patients with advanced digestive NET G3 and neuroendocrine carcinomas (NEC), which is consistent with our findings [28].

Chromogranin A (CgA) has been used as a valuable tumor marker in NETs, and elevated levels of both CgA and 5-hydroxyindoleacetic acid (5-HIAA) have previously been associated with poor prognosis in these patients [18,29]. Comparable results, especially regarding the monitoring of these markers throughout treatment, have also been documented in our research.

The immune system plays an important role in the development and progression of neuroendocrine tumors (NETs) [30]. Although the first synonym for NETs was immunologically “cold” tumors because of the reduced expression of tumor antigens and lack of stimulation of immune response, in recent years, multiple data points on the importance of immune-mediated mechanisms in the pathogenesis and outcome of patients with NET [31]. Previous investigations revealed a non-negligible role of pro- and anti-inflammatory cytokines in different biological processes of neuroendocrine tumors [32]. As cytokines can also affect and modulate the effectiveness of therapy, we investigated serum cytokine profiles in NET responders and non-responders. Our results demonstrate a significant increment in serum concentrations of IL-33 and IL-4 in non-responders in comparison to responders, while, on the contrary, sST2 was significantly higher in responders (Figure 1). Previous studies have also described an obvious correlation between IL-33 levels and tumor prognosis. Ishikawa et al. showed that IL-33 expression was significantly elevated in patients with a more severe form of squamous cell carcinoma of the tongue, pointing to the important contribution of the IL-33/ST2 axis to tumor progression [33].

IL-33 is a member of the IL-1 family of cytokines that can be involved in different pathways in immune response and tumor biology [34]. Released from destroyed and necrotic cells, IL-33 acts as an alarmin, stimulating different immune and non-immune cells [35]. The possible explanation for the increment of IL-33 may be that non-responders have an increased amount of destroyed cells able to release IL-33 that further activates innate lymphoid cells type 2 and Th2 cells as members of the type 2 immune response [36,37]. Increased levels of IL-33 and IL-4 suggested the predominance of type 2 immune response in non-responder NET patients, which may contribute to the worse therapeutic outcome and favor immune suppression [38]. Moreover, a strong positive correlation was detected between IL-33 and IL-4 in our study (Table 3). These results, together with previous findings, can support the hypothesis that by favoring type 2 immunity, increased IL-33 and IL-4 promote an immunosuppressive state followed by tumor progression [39]. On the other hand, TNF-α/IL-33, as well as TNF-α/IL-4 ratios, were significantly increased in responders compared to non-responders (Figure 1). While TNF-α is a proinflammatory cytokine, crucial for promoting a strong and functional type 1 anti-tumor immune response, IL-33 and IL-4 are associated with immunosuppression and tumor promotion [40,41,42]. Higher TNF-α/IL-33 and TNF-α/IL-4 ratios in responders suggest the predominance of proinflammatory potent stimulator TNF-α of anti-tumor immune response in NET patients who responded better to therapy and diminished immunosuppressive effect of IL-33 and IL-4. The importance of IL-33 in NET patients is supported by a positive correlation that is detected between IL-33 and disease progression (Table 3). Several studies have shown that expression of IL-33 correlates with tumor aggressiveness and poor prognosis of cancer patients [30,43]. It is possible that increased release of IL-33 contributes to less potent type 2 anti-tumor immunity and diminishes functional anti-tumor immune response. This continuous immune dysregulation may facilitate tumor progression and be associated with poor therapeutic outcomes. Moreover, a negative correlation was detected between IL-33 and neutrophil count point, indicating that elevated IL-33 may suppress neutrophil-mediated anti-tumor immune response (Table 3). As neutrophils are involved in the early response to tumors, this suppression can facilitate immune evasion and consequently worsen therapeutic response [44]. The effect of IL-33 can be neutralized in the case of the presence of the soluble ST2 (sST2) molecule, which serves as a decoy receptor for this cytokine [45]. Our results showed that systemic sST2 is significantly increased in responders in comparison to non-responders (Figure 1). The fact that increased sST2 dominates in responders, together with decreased levels of IL-33, may suggest a potential protective role of sST2 in neutralizing the pro-tumorigenic and immunosuppressive effect of IL-33, thus contributing to a better therapeutic response [46].

Importantly, we calculated a clinically relevant cut-off value for IL-33 of 146.5 pg/mL as a potential prognostic marker for therapeutic response in patients with neuroendocrine tumors. Patients with systemic levels of IL-33 above this critical value developed significantly poorer therapeutic response in comparison to patients with IL-33 levels below 146.5 pg/mL. This finding points to a possible association of increased IL-33 with a more progressive tumor phenotype or disease progression. Measuring IL-33 prior to PRRT may be helpful in stratifying patients and predicting therapeutic outcome.

## 4. Materials and Methods

### 4.1. Patient Population

This prospective, cross-sectional, single-center study included 51 patients with well-differentiated neuroendocrine tumors undergoing PRRT, which was conducted during the years 2022–2025 at the University Clinical Center Kragujevac, Serbia. All patients receiving PRRT were previously assessed, and the treatment decision was made by the NETs’ dedicated multidisciplinary tumor board. Study’s inclusion criteria, including being over 18 years of age, having a pathologically confirmed diagnosis of a neuroendocrine tumor, and demonstrating somatostatin receptor expression on diagnostic scintigraphy with 99mTc-HYNIC-TOC (Tektrotyd®, IAE POLATOM, Otwock, Poland) were met by all participants. Patients with bone marrow suppression, impaired renal function, or concurrent malignancies were omitted from this study. None of the enrolled patients were treated with antibiotics, amino salicylates, corticosteroids, statins, immunosuppressive agents, or any kind of biological therapy for at least 2 months before the beginning of this study.

The research was conducted in accordance with the Declaration of Helsinki and approved by the Ethics Committee of the University Clinical Center Kragujevac (No. 01/22-385). Written informed consent was obtained from all participants.

### 4.2. Data Collection

Data were collected from patients’ medical records and summarized in patient demographics (age, gender) and clinicopathological characteristics, including performance status (Eastern Cooperative Oncology Group (ECOG)), primary tumor location, metastatic dissemination in lymph nodes, liver, and bones, tumor grade, Ki-67 index, and tumor functionality. Tumor functionality was defined by presence of carcinoid syndrome, the most frequent functional manifestation of NETs and determined as presence of clinical symptoms related to carcinoid heart disease, diarrhea, flushing, and abdominal pain, or by elevated levels of biochemical markers such as, 24-h urine 5-hydroxyindoleacetic acid (5-HIAA), while chromogranin A (CgA) and neuron-specific enolase (*NSE*) were determined as general biomarkers [2,47].

### 4.3. Sampling

Patients’ blood was sampled on the day of admission at the Department for Nuclear Medicine, prior to the initial and before each subsequent cycle of PRRT. Complete blood count and selected biochemical analyses were performed at the Laboratory Diagnostic Service of the University Clinical Center Kragujevac. Serum cytokine levels were assessed at the Center for Molecular Medicine and Stem Cell Research, Faculty of Medical Sciences, University of Kragujevac.

### 4.4. Evaluation

#### 4.4.1. Determination of Hematological, Standard Biochemical Parameters, and Hematological/Biochemical Indices

Hematological parameters, including white blood count (WBC) and its subpopulations (neutrophils, lymphocytes, monocytes), red blood cells (RBC), hemoglobin (HGB), and platelets (PLT), were analyzed using the DxH 800 hematology analyzer.

Biochemical parameters, such as urea, creatinine, total proteins, albumin, bilirubin, alanine aminotransferase (ALT), aspartate aminotransferase (AST), gamma-glutamyl transferase (GGT), alkaline phosphatase (ALP), lactate dehydrogenase (LDH), and vitamin D, were measured with the Beckman Coulter AU5800 analyzer by spectrophotometric method. C-reactive protein (CRP) concentration was determined using the turbidimetric method with CRP Latex Beckman Coulter reagents on the same analyzer. While plasma chromogranin A and Urinary 5-hydroxyindoleacetic acid (5-HIAA) levels were determined using appropriate ELISA kits, neuron-specific enolase (NSE) was measured by an immunoradiometric assay (IRMA).

Additionally, specific hematological and biochemical indices identified as prognostic markers in cancer patients were calculated. These include the neutrophil-to-lymphocyte ratio (NLR), platelet-to-lymphocyte ratio (PLR), systemic inflammatory response index (SIRI: neutrophils × monocytes/lymphocytes), systemic immune-inflammation index (SII: neutrophils × platelets/lymphocytes), CRP-to-albumin ratio (CRP/Alb), HALP score (hemoglobin × albumin × lymphocytes/platelets), and the De Ritis ratio (AST/ALT) [19,48,49,50].

#### 4.4.2. Measurement of TNF-α, IL-4, IL-6, IL-10, IL-17, IL-33, IL-41, sST2, Galectin-1 and Galectin-3 in Sera

The concentrations of TNF-α, IL-4, IL-6, IL-10, IL-17, IL-33, IL-41, sST2, Gal-1, and Gal-3 were quantified from serum samples taken prior to the 1st PRRT cycle using commercial ELISA kits specific to human cytokines (R&D Systems, Minneapolis, MN, USA) [51].

### 4.5. PRRT and Response

All patients received at least two PRRT cycles in accordance with a standardized protocol with [177Lu]Lu-DOTATOC at a dosage of 5.5 GBq per cycle. The time interval between cycles was 8–12 weeks, consisting of a median of three treatment cycles (varying between 2 and 6 cycles). The interim staging was performed 6–8 weeks after two PRRT cycles using contrast-enhanced MDCT (Siemens SOMATOM go.Top, Siemens Healthineers, Erlangen, Germany) or MRI (Siemens Magnetom Avanto 1.5 T, Siemens Healthineers, Erlangen, Germany), depending on the initial imaging modality that was used at the time of diagnosis, as well as throughout the follow-up. All MDCT and MRI imaging examinations were conducted at the University Clinical Center Kragujevac and interpreted by experienced radiologists. Interim imaging findings were compared with those obtained before initiation of PRRT. According to the RECIST 1.1 criteria, patients were classified into two groups: responders, including stable disease (SD) and partial response (PR), and non-responders with progressive disease (PD). Patients with confirmed disease progression discontinued PRRT, given the decision of the NETs dedicated multidisciplinary tumor board [52].

### 4.6. Statistical Analysis

Statistical analyses were conducted using SPSS software for Windows (version 20.0). Continuous variables were expressed as mean ± standard error of measurement (SEM), while categorical variables were presented as proportions/percentages. To assess the normality of data distribution Kolmogorov–Smirnov and Shapiro–Wilk tests were employed. Based on the distribution characteristics, either parametric or nonparametric tests were applied (Student’s *t*-test or Mann–Whitney U test). For categorical data, the Chi-squared test was performed. Linear correlation analyses were performed using Pearson’s correlation coefficient for normally distributed data and Spearman’s rank correlation coefficient for non-normally distributed data. To evaluate the relationship between sensitivity and specificity, as well as the discriminative ability of a variable as a potential biomarker, Receiver Operating Characteristic (ROC) curve analysis was conducted. All statistical tests were performed at a 95% confidence level (CI), with *p*-values ≤ 0.05 considered statistically significant. Strength of correlation was defined as negative or positive weak (−0.3 to −0.1 or 0.1 to 0.3), moderate (−0.5 to −0.3 or 0.3 to 0.5), or strong (−1.0 to −0.5 or 0.5 to 1.0). Univariate binary logistic regression was performed to investigate the association of independent variables (initial laboratory results) with unfavorable therapeutic outcomes.

## 5. Conclusions

Taken together, the results of our study suggest that increased levels of IL-33 and IL-4 are responsible for the development of a less favorable type 2 anti-tumor immune response, which contributes to worse therapeutic response. An additional confirmation of IL-33 as a marker of poor early response in NET patients undergoing PRRT is the result that an elevated level of sST2 serves as a compensatory mechanism in responders, giving a possibility for a better therapeutic response. Our results suggest that pre-treatment measurement of IL-33 may serve as a useful biomarker for predicting poor early therapeutic outcomes in patients with neuroendocrine tumors.

## 6. Limitations

Limitations of this study are a relatively small sample size and population heterogeneity. These limitations are expected and consistent with other studies in this area, considering the low incidence of NETs and the limited number of patients eligible for PRRT. Although our study was the first to indicate the potential prognostic significance of the examined cytokines in NET patients undergoing PRRT, validation in larger and multi-center cohorts is needed to establish its clinical utility.

## Figures and Tables

**Table 1:** Demographics and clinical characteristics of NET patients.

Variables	Characteristic	Responders (n = 37)	Non-Responders (n = 14)	*p*-Value *
Age (y)		58.4 ± 2.1	56.9 ± 2.6	0.453 ^§^
Sex	Female	19 (51.4%)	10 (71.4%)	0.255 ^£^
	Male	18 (48.6%)	4 (28.6%)	
Performance status (ECOG)	0	28 (75.7%)	11 (78.6%)	0.828 ^£^
	1	9 (24.3%)	3 (21.4%)	
Location of primary tumor	GEP-NETs	25 (67.6%)	12 (85.7%)	0.366 ^£^
	Unknown primary NETs	7 (18.9%)	0 (0%)	
	Lung	3 (8.1%)	1 (7.1%)	
	Others	2 (5.4%)	1 (7.1%)	
Ki-67 (%)		17.3 ± 2.1	10.8 ± 2.1	0.076 ^¥^
Grade	G1/TC	4 (10.8%)	1 (7.1%)	0.405 ^£^
	G2/AC	25 (67.6%)	12 (85.7%)	
	G3	8 (21.6%)	1 (7.1%)	
Metastatic spread	Lymphonodal	28 (75.7%)	11 (78.6%)	1.00 ^£^
	Hepatic	34 (91.9%)	13 (92.9%)	1.00 ^£^
	Osseous	9 (24.3%)	2 (14.3%)	0.692 ^£^
Previous treatment	Surgery	15 (40.5%)	7 (50.0%)	0.715 ^£^
	Chemotherapy/targeted molecular therapies	10 (27.1%)	3 (21.4%)	
	No previous treatment	12 (32.4%)	4 (28.6%)	
Long-acting SSA	Octreotide LAR (30 mg every 4 weeks)	6 (16.2%)	3 (21.4%)	0.762 ^£^
	Lanreotide (120 mg every 4 weeks)	30 (81.1%)	11 (78.6%)	
	No	1 (2.7%)	0 (0%)	
Functionality	No	15 (40.5%)	6 (42.9%)	1.00 ^£^
	Yes	22 (59.5%)	8 (57.1%)	

Abbreviations: TC: typical carcinoid; AC: atypical carcinoid. Data represent the mean value ± SEM, frequency (percentage). * *p*-values indicate differences between responders and non-responders. ^¥^ Student’s *t*-test; ^£^ Chi-squared test; ^§^ Mann–Whitney U test. *p* < 0.05 was considered statistically significant.

**Table 2:** Laboratory parameters.

Laboratory Parameter	Before the 1st PRRT Cycle			Before the 2nd PRRT Cycle		
	Responders	Non-Responders	*p*-Value *	Responders	Non-Responders	*p*-Value *
WBC (white blood cell count) (10^9^/L)	7.08 ± 0.39	5.37 ± 0.32	**0.013** ^¥^	6.04 ± 0.32	4.83 ± 0.36	**0.04** ^¥^
Absolute Neutrophil Count (10^9^/L)	4.45 ± 0.27	3.43 ± 0.32	**0.037** ^§^	3.89 ± 0.18	3.04 ± 0.28	**0.039** ^¥^
Absolute lymphocyte count (10^9^/L)	1.95 ± 0.16	1.40 ± 0.10	**0.03** ^§^	1.51 ± 0.14	1.07 ± 0.07	**0.03** ^§^
Absolute monocyte count (10^9^/L)	0.65 ± 0.06	0.39 ± 0.04	**0.002** ^§^	0.56 ± 0.05	0.45 ± 0.10	**0.029** ^§^
RBC (red blood cell count) (10^12^/L)	4.47 ± 0.10	4.39 ± 0.12	0.479 ^¥^	4.32 ± 0.11	4.25 ± 0.14	0.792 ^¥^
Hemoglobin (g/L)	132.73 ± 2.81	122.93 ± 4.55	0.065 ^¥^	130.16 ± 2.51	123.24 ± 4.60	0.213 ^¥^
Platelets (10^9^/L)	270.73 ± 15.53	216.38 ± 22.42	**0.021** ^§^	257.73 ± 14.29	222.79 ± 45.86	**0.014** ^§^
NLR	3.55 ± 0.55	2.71 ± 0.40	0.527 ^§^	3.19 ± 0.37	3.10 ± 0.39	0.688 ^§^
PLR	205.75 ± 32.16	174.80 ± 23.81	0.883 ^§^	225.61 ± 35.32	221.86 ± 58.65	0.673 ^§^
SIRI	2.45 ± 0.57	1.11 ± 0.23	**0.021** ^§^	1.76 ± 0.21	1.07 ± 0.18	**0.049** ^§^
SII	925.55 ± 121.03	495.62 ± 77.75	**0.026** ^§^	821.69 ± 105.69	538.26 ± 78.59	**0.049** ^§^
CRP (mg/L)	6.80 ± 2.77	5.55 ± 2.66	0.499 ^§^	6.31 ± 1.69	5.72 ± 1.51	0.728 ^§^
Total protein (g/L)	70.24 ± 0.97	72.00 ± 1.07	0.241 ^¥^	69.49 ± 1.17	70.00 ± 1.60	0.983 ^§^
Albumin (g/L)	42.65 ± 0.70	42.36 ± 0.78	0.711 ^¥^	44.32 ± 0.80	42.86 ± 1.36	0.574 ^§^
CRP/Alb	0.30 ± 0.12	0.26 ± 0.13	0.506 ^§^	0.14 ± 0.04	0.15 ± 0.04	0.738 ^§^
HALP score	39.91 ± 3.77	36.58 ± 4.13	1.00 ^§^	35.25 ± 2.89	34.34 ± 4.15	0.916 ^¥^
AST (U/L)	31.49 ± 2.97	30.36 ± 4.67	0.597 ^§^	30.54 ± 2.08	48.5 ± 8.41	**0.039** ^§^
ALT (U/L)	30.84 ± 3.90	30.50 ± 7.95	0.380 ^§^	40.81 ± 9.08	46.29 ± 12.73	0.792 ^§^
De Ritis ratio (AST/ALT)	1.20 ± 0.07	1.28 ± 0.15	0.635 ^§^	1.17 ± 0.07	1.22 ± 0.15	0.975 ^¥^
GGT (U/L)	72.43 ± 11.84	56.14 ± 18.99	0.160 ^§^	77.03 ± 11.68	136.43 ± 62.84	0.387 ^§^
ALP (U/L)	100.81 ± 8.52	110.07 ± 16.28	0.435 ^§^	95.06 ± 7.59	185.75 ± 38.60	**0.029** ^§^
LDH (U/L)	415.81 ± 33.92	385.29 ± 31.99	0.941 ^§^	399.05 ± 24.19	398.00 ± 30.64	0.816 ^§^
Urea (mmol/L)	5.43 ± 0.28	5.71 ± 0.53	0.907 ^§^	5.30 ± 0.25	6.19 ± 0.42	0.053 ^¥^
Creatinine (µmol/L)	76.81 ± 2.48	88.84 ± 7.70	0.398 ^§^	76.05 ± 2.55	83.79 ± 7.52	0.650 ^¥^
eGFR (mL/min/1.73 m^2^)	86.38 ± 3.11	74.36 ± 6.30	0.102 ^¥^	87.89 ± 3.20	81.76 ± 8.56	0.250 ^¥^
CgA (ng/mL)	803.82 ± 157.21	1065.95 ± 593.78	0.399 ^§^	379.80 ± 82.92	1265.99 ± 562.86	**0.036** ^§^
5HIAA (µmol/24 h)	150.85 ± 49.74	197.47 ± 91.88	0.241 ^§^	86.35 ± 21.41	276.02 ± 106.66	**0.03** ^§^
NSE (ng/mL)	20.26 ± 8.78	18.31 ± 7.92	0.908 ^§^	12.08 ± 2.26	29.44 ± 16.06	0.250 ^§^
Vitamin D (nmol/L)	61.27 ± 4.81	40.83 ± 5.39	**0.037** ^§^	72.26 ± 5.68	48.28 ± 5.79	**0.029** ^§^

Data represent the mean value ± SEM. * *p*-values indicate differences between responders and non-responders before the 1st PRRT and before the 2nd PRRT cycle. ^¥^ Student’s *t*-test; ^§^ Mann–Whitney U test. Significant values are in bold. *p* < 0.05 was considered statistically significant.

**Table 3:** Correlation between systemic values of cytokines of interest and clinical and laboratory data.

		Spearman’s Rho	*p*-Value *
**IL-33**	**IL-4**	**0.747**	*0.001*
	**Disease progression**	**0.293**	*0.037*
	**Neutrophil Count %**	**−0.287**	*0.041*
**sST2**	**Absolute Neutrophil Count**	**0.319**	*0.022*
	**Triglycerides**	**−0.277**	*0.049*
	**Lipase**	**−0.439**	*0.001*
	**Gamma-glutamyl transferase**	**0.300**	*0.033*

Significant values of Spearman’s rank correlation coefficients are in bold and italics. * *p*-value < 0.05 was considered statistically significant.

**Table 4:** Factors associated with disease progression.

	Univariable OR	OR (95%CI)	*p*-Value *
Age (>65)	1.522	(0.4–5.791)	0.538
Sex (male)	2.368	0.628–8.926	0.203
Location of primary tumor (non-GEP-NETs)	2.880	0.554–14.960	0.208
Ki67(>10%)	4.255	1.159–15.625	**0.029**
CgA (>600 ng/mL)	1.760	0.413–7.506	0.445
NSE (>15 ng/mL)	1.145	0.297–4.424	0.844
Il/33 (>147 pg/mL)	4.552	1.159–15.628	**0.029**
IL4 (pg/mL)	1	1–1.01	0.145
sST2 (pg/mL)	1	1–1.01	0.126
TNF (pg/mL)	0.99	0.994–1.004	0.621

Univariate binary logistic regression was performed, and significant values are shown in bold. * *p*-value < 0.05 was considered statistically significant.

## Data Availability

All data and materials supporting this study will not be publicly available due to ethical restrictions. The data will be available upon reasonable request from the corresponding author.
